# Antarctic *Streptomyces fildesensis* So13.3 strain as a promising source for antimicrobials discovery

**DOI:** 10.1038/s41598-019-43960-7

**Published:** 2019-05-16

**Authors:** Kattia Núñez-Montero, Claudio Lamilla, Michel Abanto, Fumito Maruyama, Milko A. Jorquera, Andrés Santos, Jaime Martinez-Urtaza, Leticia Barrientos

**Affiliations:** 1 Laboratorio de Biología Molecular Aplicada, Centro de Excelencia en Medicina Traslacional, Universidad de La Frontera, Temuco, Chile; 2 Núcleo Científico y Tecnológico en Biorecursos (BIOREN), Universidad de La Frontera, Temuco, Chile; 3 Centro de Investigación en Biotecnología, Escuela de Biología, Instituto Tecnológico de Costa Rica, Cartago, Costa Rica; 4 Department of Microbiology, Graduate School of Medicine, Kyoto University, Yoshida‒Konoe‒cho, Sakyo‒ku, Kyoto, Japan; 5 Laboratorio de Ecología Microbiana Aplicada, Departamento de Ciencias Químicas y Recursos Naturales, Universidad de La Frontera, Temuco, Chile; 6 Centre for Environment, Fisheries and Aquaculture Science (CEFAS), Barrack Road, Weymouth, Dorset DT4 8UB UK

**Keywords:** Antibiotics, Bacterial genes, Applied microbiology, Bacteriology, Genome evolution

## Abstract

Antarctic have been suggested as an attractive source for antibiotics discovery and members of *Streptomyces* genus have historically been studied as natural producers of antimicrobial metabolites. Nonetheless, our knowledge on antibiotic-producing *Streptomyces* from Antarctic is very limited. In this study, the antimicrobial activity of organic extracts from Antarctic *Streptomyces* strains was evaluated by disk diffusion assays and minimum inhibitory concentration. The strain *Streptomyces* sp. So13.3 showed the greatest antibiotic activity (MIC = 15.6 μg/mL) against Gram-positive bacteria and growth reduction of Gram‒negative pathogens. The bioactive fraction in the crude extract was revealed by TLC‒bioautography at R_f_ = 0.78 with molecular weight between 148 and 624 m/z detected by LC-ESI-MS/MS. The strain So13.3 was taxonomically affiliated as *Streptomyces fildesensis*. Whole genome sequencing and analysis suggested a 9.47 Mb genome size with 42 predicted biosynthetic gene clusters (BGCs) and 56 putative clusters representing a 22% of total genome content. Interestingly, a large number of them (11 of 42 BGCs and 40 of 56 putative BGCs), did not show similarities with other known BGCs. Our results highlight the potential of the Antarctic *Streptomyces* strains as a promising source of novel antimicrobials, particularly the strain *Streptomyces fildesensis* So13.3, which first draft genome is reported in this work.

## Introduction

Historically, most of bioactive compounds used in medicine are originally derived from natural sources, such as microorganisms. In this context, *Streptomyces* is a bacterial genus widely studied as a source of antimicrobial secondary metabolites^1^ where 80% of the known antibiotics are from *Streptomyces*^2^. The members of *Streptomyces* genus are Gram‒positive filamentous bacteria characterized by large genomes, ranging from 6.2 Mb to 12.7 Mb^3,4^. Studies have revealed that genomes of *Streptomyces* can harbor a wide battery of biosynthetic gene clusters (BGCs) responsible for the production of secondary metabolites with antimicrobial activity, including polyketide synthases (PKS), non‒ribosomal peptide synthetases (NRPS), bacteriocins^5^, among others. However, despite the growing number of studies reporting the exponential increase of antimicrobial compounds from *Streptomyces* during last decades, the discovery and identification of novel antibiotics have been very limited^6,7^.

In nature, bacterial secondary metabolites play an important ecological and physiological role in the interactions and processes within microbial communities (e.g., colonization and stress response)^8,9^. Contributions of secondary metabolites are still more relevant under extreme conditions, where bacteria have evolved and developed strategies to survive and proliferate under adverse circumstances^10^, including the acquisition of unique chemically complex pathways for the synthesis of secondary metabolites^11^. Antarctica is a unique, pristine and extreme environment, considered as the coldest, driest, and windiest place on the globe^12^ which remains poorly explored. Therefore, Antarctic bacteria have been proposed as a promising source for novel antimicrobial secondary metabolites^13^. Recent genomic data analyses have revealed the presence of unique adaptation mechanisms, metabolic pathways, and secondary metabolites in Antarctic bacteria, such as *Pseudoalteromonas* sp.^14–16^. However, our knowledge of functional and genomic information of antimicrobial‒producing bacteria in the Antarctic ecosystem remains extremely limited, which is particularly relevant for new species of the genus *Streptomyces* as a potential source of secondary metabolites with antimicrobial activity.

With this aim in mind, we examined the antimicrobial activity of *Streptomyces* strains isolated in the Antarctica and selected particular strain, *Streptomyces* sp. So13.3, to investigate the production of secondary metabolites with antimicrobial potential by genome sequencing and identification of BCGs encoding for potential antimicrobial compounds.

## Methods

### Cultures of Streptomyces strains

In a previous report Antarctic *actinobacteria* strains were isolated and identified^17^. Eight *Streptomyces* strains from our previous work were used in this study (Table 1). Stock cultures were first grown onto M1 agar plates (peptone 2 g/L, yeast extract 4 g/L, starch 10 g/L, and agar 18 g/L; pH 7.0) at 15 °C for one week. Stock cultures were then used to inoculate 25 mL of M1 broth, which were incubated at 15 °C for 5 days under shaking (120 rpm) and used as starter cultures. Starter cultures were used to scale toward larger volume cultures (150, 250, 1000 and 2000 mL) required to obtain enough amounts of organic and DNA extracts for antimicrobial and genomic analysis, respectively. Similar to starter cultures, larger volume cultures of *Streptomyces* strains were grown in M1 broth and incubated at 15 °C for one week under shaking (120 rpm).

**Table 1:** *Streptomyces* strains isolated from Antarctic soils and used in this study.

Strain	Sampling site	Sampling year	Closest relatives or cloned sequences^a^
So1	Fildes Peninsula, King George Island	2014	*Streptomyces* sp.^17^
So13.3	Fildes Peninsula, King George Island	2014	*Streptomyces* sp.^17^
Decept/INACH3013	Fildes Peninsula, King George Island	2011	*Streptomyces fildesensis* ^49^
So1C	Fildes Peninsula, King George Island (25 de Mayo) (ASPA #125)	2014	*Streptomyces thermospinosisporus* ^17^
So5.1	Byers Peninsula, Livingston Island, South Shetland Islands (ASPA #126)	2014	*Streptomyces* sp.^17^
So64.7	Ardley Island, Maxwell Bay, King George Island (25 de Mayo) (ASPA #150)	2014	*Streptomyces fildesensis* ^17^
So64.6	Ardley Island, Maxwell Bay, King George Island (25 de Mayo) (ASPA #150)	2014	*Streptomyces beijiangensis* ^17^
So3.2	Coppermine Peninsula, Robert Island, South Shetland Islands (ASPA #112)	2016	*Streptomyces luridus* ^95^

^a^Based on partial sequencing of 16S rRNA and comparison with those present in GenBank database from NCBI by using BLASTN (http://www.ncbi.nlm.nih.gov/blast) as previously described^17,49^.

ASPA: Antarctic Special Protected Area.

### Extraction of organic extracts from cultures of Streptomyces strains

Organic crude extracts from bacterial cultures were obtained by solvent extraction with ethyl acetate. Ethyl acetate was chosen because it has been previously reported to be suitable for extraction of antimicrobial metabolites from *actinobacteria*^18,19^. The mixtures of crude culture:solvent (1:1 ratio) were vigorously shaken for 10 min and kept stationary from 15 to 30 min until separation of aqueous and organic phases. Organic phases were collected and concentrated in a rotary evaporator (model RE100‒Pro; SCILOGEX, LLC, CT, USA) at 40 °C and 80 rpm. The final concentrated extracts (approximately 2 mL) were transferred to pre‒weighed tubes and dried at room temperature for at least 24 h. The dried crude extracts were then weighed, dissolved in 200 μL of methanol, and finally stored at −20 °C until assay their antimicrobial activity.

In addition, the amount of crude extracts output by each *Streptomyces* strain and calculation of growth kinetics was estimated as an additional characterization parameter for each strain. Briefly, culture samples (1 mL) were taken from bacterial cultures every 24 h and the absorbance (600 nm) was measured with a spectrophotometer (Optizen Pop UV/Vis; Mecasys Co., Ltd.; Daejeon, Korea). Specific growth rate (μ) was calculated with the following Eq. (1):

1 $$\mu =\frac{dx}{dt}\cdot \frac{1}{X}$$

where, µ = specific growth rate (h^−1^); *x* = 600 nm absorbance on exponential growth; *t* = time (h). The biomass, measured as dry cell weight (g/L), was determined during the stationary phase of bacterial growth. Samples in triplicate (1 mL) were deposited on a pre‒weighed 1.5 mL microtube and dried at 100 °C to obtain a constant weight.

### Screening of antibacterial activity of organic extracts from Streptomyces strains

Concentrated organic crude extracts obtained in Section 2.2 were screened for their inhibitory activity against 12 known pathogenic bacterial strains by disk diffusion assay (DDA) as established by the Clinical & Laboratory Standards Institute (CLSI)^20,21^. Briefly, 15 µL of organic crude extract at 10 mg/mL was added to 6 mm‒Oxoid^TM^ Blank Antimicrobial Susceptibility Disks (Thermo Fisher Scientific Inc., Waltham, MA, USA) and dried for one hour at room temperature. Each disk was then placed onto Muller‒Hinton agar plates (BD Difco^TM^, Becton, Dickinson and Company, NY, USA) previously plated with a suspension of test pathogenic bacteria at 0.5 McFarland turbidity (equivalent to 1.5 × 10^8^ cells/mL). Diameters of inhibition zones were measured after 20 h of incubation at 37 °C. Tested pathogens included three bacterial strains from the American Type Culture Collection (ATCC) (*Escherichia coli* ATCC 22925, *Staphylococcus aureus* ATCC 25923 and *Klebsiella pneumoniae* ATCC 13883) and seven strains from the Chilean Collection of Type Cultures (CCCT) previously isolated from local clinical samples (*Acinetobacter baumannii* CCCT 18.3, AmpC β‒lactamase*-*producing *Escherichia coli* CCCT 18.4, *Salmonella paratyphi* CCCT 18.5, carbapenem-resistant *Klebsiella pneumoniae* CCCT 18.6, *Pseudomonas aeruginosa* CCCT 18.7, *Enterococcus* sp. CCCT 18.8, *Enterococcus faecalis* CCCT 18.9, and methicillin‒resistant *Staphylococcus aureus* CCCT 18.10 or MRSA).

### Antimicrobial activity and chemical characterization of selected crude extracts from Streptomyces strains

Three organic crude extracts that showed the greatest antimicrobial activity by DDA were selected and their inhibitory activity was newly tested to determine the Minimum Inhibitory Concentration (MIC) by microdilution assay as described in the CLSI standards^22,23^. Briefly, concentrated crude extracts were serially diluted (500, 250, 125, 62.5, 31.25, and 15.625 μg/mL) in Muller‒Hinton broth (BD Difco^TM^) and distributed in triplicate in 96‒microwell plates at a final volume of 100 μL. Test pathogenic bacteria -same strains included in DDA assay- were inoculated (approximately 5 × 10^5^ CFU/mL) in each microwell, homogenized by mixing, and incubated at 37 °C for 20 h. Microwells with Muller‒Hinton broth without crude extracts were used as controls. Nalidixic acid was included as positive and quality control (8, 4, 2, 1, 0.5, and 0.125 μg/mL). The absorbance (625 nm) was measured before and after incubation to detect differential growth of the microorganisms exposed to different dilutions of crude extracts.

Based on the lowest MIC observed, concentrated organic crude extract from *Streptomyces* So13.3 was chosen and used to characterize the bioactive fraction by thin layer chromatography (TLC). TLC technique is suitable for the separation of organic compounds in complex mixtures by chromatography and allows the direct identification of the compound with antimicrobial activity by studying their effects on test microorganisms^18,24^. A spot of the crude organic extract was loaded onto TLC silica gel aluminum sheets (Merck KGaA, Darmstadt, Germany) and separation of compounds in the organic extract was tested with the following mobile phases: dichloromethane:ethyl acetate (1:9 and 2:8), methanol:ethyl acetate (1:9 and 2:8), and hexane:ethyl acetate (2:8). After running TLC, plates were dried for at least 1 h and then separated spots were visualized with sprayed ninhydrin reagent or projection of UV light onto the plates, and retardation factor (R_f_) for each spot was calculated. Mobile phase that generates greater separation of the compounds was used for bio‒autography assays. With sterile forceps, dried TLC plates with separated compounds were placed on a Muller‒Hinton agar plate previously inoculated with a suspension of the pathogens *S*. *aureus* ATCC 25923 and MRSA at 0.5 McFarland turbidity. A TLC loaded with methanol (used for dissolving the crude extract) was included as negative control. The agar plates were pre‒incubated at 4 °C for 1 h to allow the diffusion of compounds into the agar. Afterwards, TLC plates were removed, and agar plates were incubated at 37 °C for 20 h. Fractions of crude extract with antibacterial activity were revealed by inhibition zones.

Active crude extract methanol active fraction was analyzed by HPLC-ESI-MS/MS in a Mass Applied Biosystems/MDS Sciex 3200 Qtrap instrument with Electrospray Turbo VTM ionization source using Analyst 1.5.1 software. Aliquots were directly injected with a Harvard syringe pump at 10 µl/min. Parameters were set on positive mode, 20 psi Cur gas, High CAD gas, 13 psi Gas1, and 5500 V ionization. Method EMS (Enhanced Mass Scan) was selected for a general scan in 100–1000 m/z mass range at 1000 Da/s. Selected ions were scanned with Enhanced Product Ion (EPI) method with 50–1000 m/z mass range at 4000 Da/s. ESI-MS/MS acquired data were subjected to molecular networking analysis generated in Global Natural Products Social Molecular Networking (GNPS) server^25^ using the spectral clustering analysis. A cosine score of 0.5 and a minimum number of match peaks of 6 were selected for this analysis. The spectral networks were imported and visualized using Cytoscape 3.7.0 software.

### Whole genome sequencing of Streptomyces sp. So13.3 and search of putative antimicrobial gene clusters in the genome

Whole genome sequencing of the *Streptomyces* sp. So13.3 was performed to investigate its antimicrobial potential based on biosynthetic gene clusters (BGCs) encoding secondary metabolites. Genomic DNA extraction was performed with UltraClean Microbial DNA Extraction Kit (Mo Bio Laboratories, Carlsbad, USA). Paired-end libraries with average insert size of 350-bp were prepared, followed by 2 × 150-bp sequencing on Illumina HiSeq X ten sequencing platform. The quality of the reads were determined using FastQC^26^ (see quality report in Supplementary Fig. S5) and filtered with Trimmomatic 3.0^27^. Potential contamination of the reads were assessed using Automated Contamination Detection and Confidence Estimation (ACDC)^28^ and filtering unmapped reads by Burrows‒Wheeler Alignment tool^29^ with *mem* algorithm and the following parameters: band width = 10000, matching score = 1, mismatch penalty = 1, gap open penalty = 1, gap extension penalty = 0, clipping penalty = 1, penalty for an unpaired read pair = 0, and output alignment min score = 1.

*De novo* assembly was performed using SPAdes 3.11.1 with default parameters and *k-mer* = 21, 33, 55, 77^30^, Velvet 1.2.10^31^ with default parameters for short reads and *k-mer* = 21, 25, 27, 31, and A5‒Miseq with default parameters^32^. The best assembly was selected based on the number of contigs and N50. The draft genome was used as input to CheckM (default options)^33^ to determine its quality regarding completeness and contamination. The genome annotation was accomplished using NCBI Prokaryotic Genome Annotation Pipeline (released 2013)^34^. The tool antiSMASH 4.0.2.^35^ with default parameters and all features selected, was employed to identify biosynthetic gene clusters (BGCs) and putative BGCs encoding secondary metabolites. The image genome visualization was produced by DNAplotter 10.2^36^. This Whole Genome Shotgun project has been deposited at DDBJ/ENA/GenBank under the accession PYSU00000000. The version described in this paper is PYSU01000000.

Molecular identification of *Streptomyces* sp. So13.3 was performed by phylogenetic analysis based on rRNA 16S complete gene sequence subtracted from draft genome. The nearest taxonomic group was identified by 16S rDNA nucleotide sequence BLASTN (http://www.ncbi.nlm.nih.gov/blast) using DDBJ/EMBL/GenBank nucleotide sequence databases. Closest rRNA 16S sequences were used for the phylogenetic tree construction using Mafft alignment version 7^37^ L-INS-i method. The alignment file was used to build a Maximum Likelihood (ML) phylogenetic tree. The most accurate substitution model was estimated using Model Finder method with the Akaike Information Criterion^38^ using IQ-TREE web server^39^. Phylogenetic tree was constructed in SeaView version 4^40^ using ML with GTR substitution model, 1000 bootstrapped data sets, 4 substitution rate categories for across site rate variation, estimated gamma distribution parameter, optimized variable sites and empirical nucleotide equilibrium frequencies. Starter trees for the heuristic search were obtained by BioNJ algorithms, and tree topology search was performed with NNIs.

Additionally, a microbial genome BLAST was performed using the draft genome from *Streptomyces* sp. So13.3 as query against *Streptomyces* taxid: 1883 by megablast program. Comparative whole genome analysis was carried out between the 22 closest *Streptomyces* strains obtained from the genome BLAST (higher identity percent) using progressive Mauve^41^ for alignment and phylogenetic tree construction to estimate the genetic relatedness between the species. Accession numbers for the genomes of the closest *Streptomyces* species were as follows: NZ_CP017157.1, GCA_003851665.1, FNST01000002.1, NZ_CP015588.1, NZ_CP011533.1, NZ_AJGV00000000.1, NZ_LIQY00000000.1, NC_003155.5, NZ_CP011340.1, NC_016582.1, NZ_JOFL00000000.1, GCF_000383595.1, NC_003888.3, NZ_CP013129.1, NZ_CP016279.1, NZ_CP010407.1, NZ_GG657757.1, RJKZ01000001.1, NZ_JQNR00000000.1, NZ_GG657754.1, NZ_JNWJ01000004.1, NZ_CP016438.1. BGCs were also predicted for the selected *Streptomyces* species as described above, and compared using iTOL v3 visualization tool^42^.

## Results

### Antimicrobial activity in crude extracts from Streptomyces strains

Growth kinetics of the different *Streptomyces* isolates are described in Supplementary Fig. S1, the stationary phase of microbial growth was usually reached at the fourth and fifth day of culture, while the calculated specific growth rate (μ) ranged from 0.08 h^−1^ to 0.18 h^−1^ in M1 broth. These results are similar for other Streptomyces members previously reported, including *S*. *coelicolor* (0.02 h^−1^ to 0.30 h^−1^)^43^, *S*. *albogriseolus* (0.20 h^−1^)^44^, *S*. *clavuligerus* (0.03 h^−1^ to 0.1 h^−1^)^45^. The higher specific growth rate was showed by So13.3 strain, expectedly being more efficient in growth yield^46^. Organic crude extracts from bacterial cultures were obtained by ethyl acetate solvent extraction, with ranges from 1.74 (*Streptomyces* sp. So3.2) to 3.99 (*Streptomyces* sp. So13.3) mg per gram of cells. The results revealed that all crude extracts from *Streptomyces* strains showed antimicrobial activity by DDA against assayed Gram‒positive pathogenic bacteria, but no inhibition activity on Gram‒negative pathogenic bacteria was observed (Table 2). Similar results have been reported for *Streptomyces* strains and other *actinobacteria* strains from Antarctic soils^47–49^, which exhibited antibacterial activity against Gram‒positive pathogenic strains. The crude extract from *Streptomyces* sp. So13.3 produced the greatest inhibitory activity against Gram‒positive pathogenic bacteria among the Antarctic strains, particularly for *Enterococcus* sp. and *E*. *faecalis*. The antibacterial activity of *Streptomyces* sp. So13.3 was followed by *Streptomyces* sp. So64.6 and *Streptomyces* sp. So3.2.

**Table 2:** Diameters of inhibition zones on agar plates of test pathogenic microorganisms exposed to concentrated crude extracts from Antarctic *Streptomyces* strains.

Pathogenic microorganisms	Diameter of inhibition zones (mm)/MIC (μg/mL)*							
	So13.3	So64.6	So3.2	Decept	So1C	So5.1	So64.7	So1
***Gram-positive strains***								
*Salmonella paratyphi* CCCT 18.5	7/15.6	9/>500	9/>500	7/nd	8/nd	−/nd	7/nd	8/nd
*Enterococcus sp*. CCCT 18.8	14/15.6	8/>500	8/>500	7/nd	7/nd	−/nd	−/nd	−/nd
*Enterococcus faecalis* CCCT 18.9	15/15.6	8/>500	−/>500	7/nd	−/nd	8/nd	−/nd	−/nd
*Staphylococcus aureus* ATCC 25923	10/15.6	7/>500	7/>500	9/nd	7/nd	−/nd	−/nd	−/nd
*Staphylococcus aureus* CCCT 18.10	11/15.6	8/>500	8/>500	−/nd	−/nd	7/nd	−/nd	−/nd
***Gram- negative strains***								
*Acinetobacter baumannii* CCCT 18.3	−/>500	−/>500	−/>500	−/nd	−/nd	−/nd	−/nd	−/nd
*Klebsiella pneumoniae* ATCC 13883	−/>500	−/>500	−/>500	−/nd	−/nd	−/nd	−/nd	−/nd
*Klebsiella pneumoniae* CCCT 18.6	−/>500	−/>500	−/>500	−/nd	−/nd	−/nd	−/nd	−/nd
*Pseudomonas aeruginosa* CCCT 18.7	−/>500	−/>500	−/>500	−/nd	−/nd	−/nd	−/nd	−/nd
*Escherichia coli* ATCC 22925	−/>500	−/>500	−/>500	−/nd	−/nd	−/nd	−/nd	−/nd
*Escherichia coli* CCCT 18.4	−/>500	−/>500	−/>500	−/nd	−/nd	−/nd	−/nd	−/nd

ATCC: American Type Culture Collection; CCCT: Chilean Collection of Type Cultures; (−): no inhibitory effect.

nd: not determined.

*MIC values were determined up to 500 μg/mL, extracts that did not show inhibition at this higher tested concentration are reported as >500 μg/mL.

### Antimicrobial activity and chemical characterization of selected organic extracts from Streptomyces strains

The potential of antibacterial activity of organic crude extracts was estimated by MIC using the *Streptomyces* strains that showed the higher inhibitory activities among the Antarctic isolates on agar plate by DDA technique (So13.3, So64.6 and So3.2). The MIC values were found to be 15.6 μg/mL for So13.3 against Gram-positive bacteria, while MIC value for Gram-negative bacteria was not reached at 500 μg/mL (Table 2). As well, the MIC value for strains So64.6 and So3.2 showed to be >500 μg/mL (Table 2). It is noteworthy that despite the fact that *Streptomyces* sp. So13.3 did not show inhibitory activity on the agar plate by DDA, MIC analysis revealed that *Streptomyces* sp. So13.3 extract does not inhibit the growth of Gram-negative bacteria, but it significantly reduced the growth of pathogenic *E*. *coli* ATCC 22925, *E*. *coli* CMY‒2 and *A*. *baumannii* (see Supplementary Fig. S2). This result suggest that higher concentrations of So13.3 extract could show an inhibition of Gram-negative pathogens, which was not observed on DDA assay because each disk concentration (1.5 μg/mL) is far below the MIC value of So13.3 extract for Gram-negative bacteria (>500, μg/mL). Comparably, other authors have screened *Streptomyces* sp. purified extracts for antimicrobial activity (ε‒poly‒l‒lysine and a diketopiperazine derivative), and usually higher MIC values were reported for Gram‒negative bacteria^50,51^. Additionally, extracellular compounds from *S*. *lividans* have been found to change the behavior of Gram‒negative bacteria^52^. MIC of 4 μg/mL of a purified compound is recommended as susceptible breakpoint to inhibit clinical pathogenic microorganisms^23^; therefore, due to the higher antimicrobial activity (measured as DDA and MIC) and the higher growth rate (see Supplementary Fig. S1) of *Streptomyces* sp. So13.3 compared to other *Streptomyces* strains, this strain was selected for a more detailed exploration at genomic and functional level.

**Figure 1:**
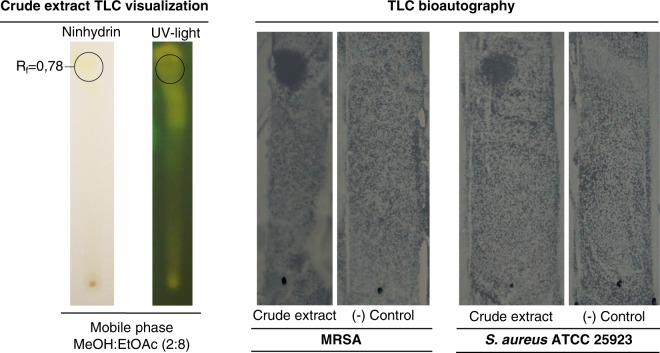
Thin layer chromatography (TLC) showing organic fraction with antimicrobial activity from *Streptomyces* sp. So13.3 crude extract against MRSA and *S*. *aureus* ATCC 25923. TLC was revealed by spraying with ninhydrin reagent or UV light exposition and TLC‒bioautography assay on MRSA and *S*. *aureus* ATCC 25923 is shown. Active fraction of the crude extract in the TLC is shown with a circle selection (retardation factor (R_f_) = 0.78).

**Figure 2:**
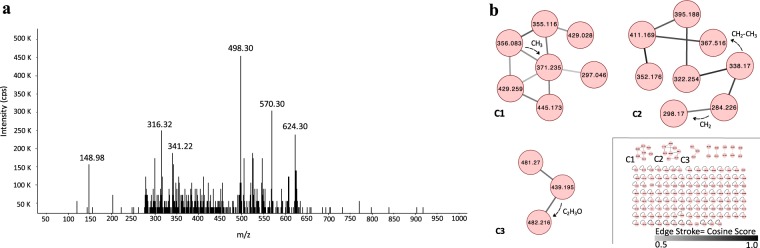
LC-ESI-MS/MS data from *Streptomyces fildesensis* So13.3 crude extract. (**a**) MS/MS spectrum showing the mass of main compounds in the crude extract. (**b**) Dereplication and Molecular Networking analysis of MS/MS data via the Global Natural Products Social Molecular Networking (GNPS). Line stroke between nodes (metabolites) describes the similarity of the linked parent masses spectra. Dashed arrows show presumed structural modifications from each node based on mass differences.

The characterization of the bioactive compounds of the crude extracts of *Streptomyces* sp. was carried out by means of TLC bio-autography and mass spectrometry (LC-ESI-MS/MS). The mobile phase used for TLC was the mixture methanol:ethyl acetate (2:8), which allowed the visualization of a spot of antimicrobial organic compounds (Rf: 0.78) (Fig. 1). This result demonstrates that one compound or mixture of compounds with similar polarity are responsible for the antimicrobial activity, a pattern which was confirmed to be the same for *S*. *aureus* ATCC 25923 and MRSA pathogenic strains. Coincidently, similar analyses with different *Streptomyces* strains have shown active compounds with R_f,_ ranging from 0.11 to 0.86, where the upper organic phase corresponded to antimicrobial molecules, such as streptoclorine, nigericin and piericidin A1^53–55^. In addition, the result of the LC-ESI-MS/MS revealed an approximation of the molecular weight of the antibiotics produced by *Streptomyces fildesensis* So13.3. As expected MS/MS data showed the detection of multiple metabolites on the crude extract, with higher intensity at molecular weights of 498.30 and 570.30 m/z (Fig. 2a), but peaks were also obtained between 148 and 624 m/z. Metadata analysis from MS/MS spectra by GNPS showed that 145 metabolites were detected in the crude extract (precursor masses range from 109.99 to 624.30 m/z). No matches were found with known metabolites from GNPS natural compounds library. Three molecular networks composed of three or more molecules were revealed by molecular networking (Fig. 2b). Each network is a spectral correlation and visualization that detects sets of spectra from structurally related molecules, even when the spectra themselves did not matched to any known compound, where each spectrum is presented as a node, and spectrum-to-spectrum alignments as edges (connections) between nodes^56^. The found networks are composed of eight, seven and three nodes, respectively, which represents molecules from the same molecular family. Chemical and structural differences from nodes into the molecular families can be presumed based on mass differences, as shown in Fig. 2b where m/z differences between two related nodes match with methyl, methylene and acetyl groups. This approximation has been reported for the identification of novel drugs and analogs from known metabolites^57–60^.

### Genome analysis, taxonomic affiliation and identification of antimicrobial gene clusters in Streptomyces sp. So13.3 genome

**Figure 3:**
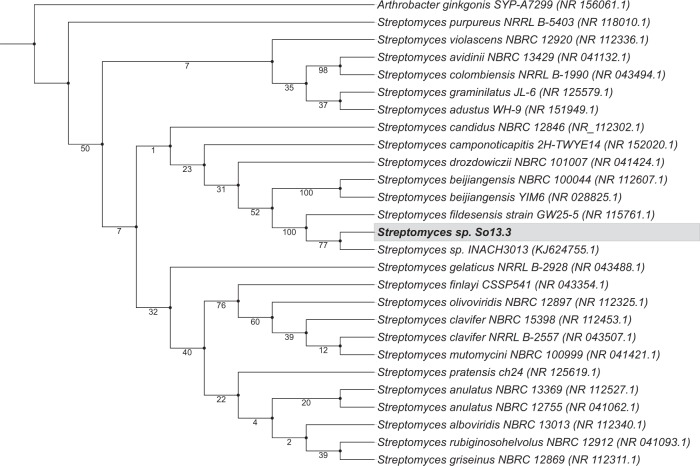
Maximum-likelihood phylogenetic tree based on complete 16S rRNA gene sequences showing the genetic distances between strain *Streptomyces* sp. So13.3 and closely related reference species. *Arthrobacter ginkgonis* SYP-A7299 16S rRNA gene sequence was included as the outgroup. Numbers at nodes represent the bootstrap support (%). Accession number of 16S rRNA gene sequences are shown in brackets.

Draft genome sequences assembly was performed with three different tools, with better results obtained using SPAdes 3.11.1 with *Kmer* size 77. Assembly resulted in a linear chromosome with a size of 9.47 Mb and 70.47% GC content (Table 3). By subtraction of complete 16S rRNA gene sequence and phylogenetic analysis this strain was identified as *Streptomyces fildesensis* species (Fig. 3). *S*. *fildesensis* was first described in^61^ as a novel *Streptomyces* species isolated from Antarctic soil and antimicrobial activity from a close related strain has been reported^49^. Annotation reveals that genome of *S*. *fildesensis* So13.3 consist of 8077 genes, 71 transfer RNAs (tRNA) and 17 ribosomal RNA (rRNA). In general, these results are in agreement with the characteristics of *Streptomyces* genomes previously reported^62^. Therefore, our assembled sequences provide a high-quality draft genome with less than 3% contamination and 98.68% completeness, corresponding to a first approach to studying the antimicrobial potential of *S*. *fildesensis* sp. So13.3 Antarctic strain by means of genomics analysis.

**Table 3:** Statistical summary of results obtained from draft genome sequences assembly of *Streptomyces* sp. So13.3.

Statistic parameter	*Streptomyces fildesensis* So13.3	Range for *Streptomyces* spp^a^
Genome size	9,475,060 bp	6.84~11.94 Mb
Number of scaffolds	227	100~200
Largest contig^b^	319,501	—
N50^b^	103,702	—
L50^b^	29	—
GC (%)^b^	70.47	70.6~73.3
Number of ncRNA^c^	3	1–3
Number of rRNA^c^	17	6~21
Number of tRNA^c^	71	64~80
Number of CDS^c^	8,077	6,331~9,309
CRISPR arrays	3	—

^a^Based on 15genomes^62^.

^b^Statistic based on contigs of ≥500 bp.

rRNA: ribosomal rRNA; tRNA: transfer RNA; CDS: coding sequences.

**Figure 4:**
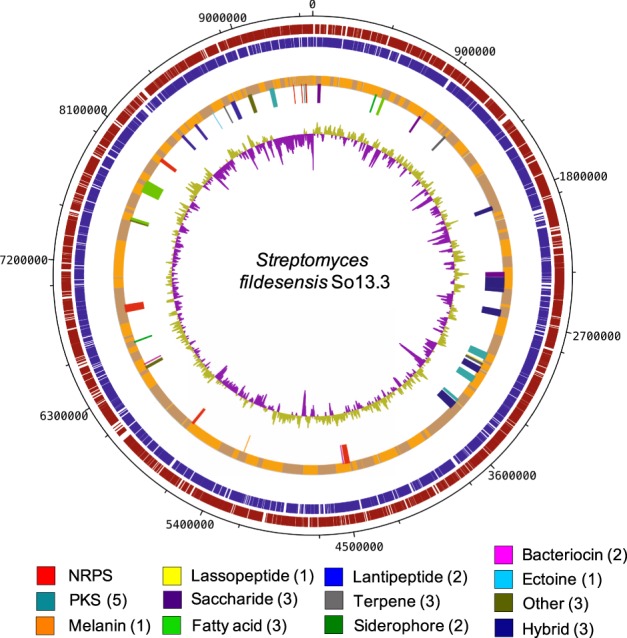
Secondary metabolite gene clusters predicted in *Streptomyces fildesensis* sp. So13.3 genome. Representation *S*. *fildesensis* So13.3 genome characteristics. From outside inward: DNA strands reverse and forward; contigs, secondary metabolites gene clusters (BGCs) and GC content. BGCs types are shown with different colors according to the legend. “Others” refers to clusters for secondary metabolites that do not fit the standard classifications.

**Figure 5:**
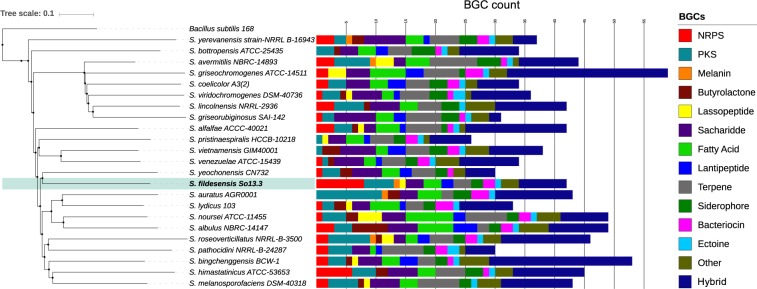
Phylogenetic tree based on complete genome sequences showing the genetic distances between *S*. *fildesensis* So13.3 strain (highlighted in light blue) and closely related reference species. Bars chart (right) shows the number of BGCs per *Streptomyces* species, with different colors denoting different BGC types. *Bacillus subtilis* 168 (Accession NC_000964.3) was included as the outgroup. Tree scale bar = branch length.

Forty‒two putative gene clusters for secondary metabolites were detected in the genome of *S*. *fildesensis* So13.3, including diverse antimicrobials (Fig. 4). Most of the BGCs corresponded to non‒ribosomal peptide‒synthetase (NRPS; 8 of 42), type I and II polyketide synthase (PKS; 5 of 42) and hybrid types (8 of 42), but lantipeptides, terpenes, bacteriocins, melanin, lassopeptides, siderophores, ectoine, saccharides, fatty acids and butyrilactone gene clusters were also found (Fig. 4, Supplementary Table S1). All previous types have been studied for their antimicrobial potential, but particularly non‒ribosomal peptide synthetase (NRPS) and polyketide synthase (PKS I and II) pathways, which are thought to be responsible for antibiotic synthesis in *actinobacteria*^63^. The number of BGCs of secondary metabolites observed is higher than the average usually reported for other mesophilic *Streptomyces* strains, with ranges from 20 to 30 gene clusters^62,64,65^, with exceptions such as *Streptomyces* strains isolated from Indonesia^66^ and China^67^, which had more than 50 clusters for secondary metabolites. As suggested for Antarctic *Pseudoalteromonas* strains ‒with a maximum of 19 biosynthetic gene clusters‒^16^, it have been thought that a greater number of secondary metabolites could be driven by the adaptation and evolution of *Streptomyces* strains to survive and proliferate under harsh Antarctic conditions. In this study, we additionally include an updated report of BCGs found in 22 *Streptomyces* sp. genomes belonging to the closest strains to *S*. *fildesensis* So13.3 based on Genome Blast identity (Fig. 5). The results showed that those *Streptomyce*s strains share a high BGCs abundance in their genomes, with a mean of 40 BGCs per strain ranging from 26 (*S*. *pristinaespiralis* HCCB‒10218) to 59 (*S*. *griseochromogenes* ATCC‒14511). No evident differences or patterns were found on BGCs composition and abundance between the clades obtained by the phylogenetic analysis (Fig. 5), suggesting that secondary metabolites clusters were acquire by horizontal gene transference (HGT) in *Streptomyces*. Also, the variable gene cluster compositions and abundance inner clades with high similarity suggest that BGCs composition could not be assumed from taxonomic identification even when based on genomic approaches. This information implies that different potential bioactive secondary metabolites could be discovered from close related *Streptomyces* strains.

To our knowledge, this is the first draft genome report from an Antarctic *Streptomyces* strain and for *S*. *fildesensis* species aimed at elucidating its antimicrobial potential. Despite the bioinformatics analysis that suggested the presence of 42 gene clusters encoding secondary metabolites, only 4 clusters could be identified containing 100% of the genes from the known cluster for ectoine, SapB, alkylresorcinol and 2‒methylisoborneol biosynthetic clusters (see Supplementary Fig. S3), which are frequently found in *Streptomyces* strains^64,66,68,69^. Ectoine is an osmolite proposed to provide protection against osmotic stress commonly found in *Streptomyces* strains^70,71^, SapB is a type III lantipeptide produced during the aerial mycelium formation process with biosurfactant activity^72,73^, 2‒methylisoborneol is a volatile organic compound commonly found in terrestrial and aquatic microorganisms^74–76^ with antimicrobial activity at determined concentrations^77^ and alkylresorcinol is a type III PKS previously described in *Streptomyces griseus* with multiple and not completely understood biological functions^78,79^. None of the above compounds have been reported as strong antimicrobials. Nonetheless, the gene clusters identified on *S*. *fildesensis* So13.3 contain other genetic elements, related with different biosynthetic pathways, regulation or transport of those metabolite which could be producing modified metabolites (Supplementary Fig. S3). On the other hand, some of the clusters (17%) showed high gene similarity (>50%) with the known metabolites including the antibiotics actinomycin, lysolipin, the anticancer molecule marineosin, and the protease/kinase/phosphatase inhibitor RK-682. Despite this, most of the clusters (48%) displayed low similarity (<50) or did not match (26%) with other known secondary metabolites (Supplementary Table S4), suggesting the potential of *S*. *fildesensis* So13.3 as source of novel bioactive compounds, including antibiotics.

Comparison of AntiSMASH analysis from the nearest genomes to *S*. *fildesensis* So13.3 showed that, although our strain exhibit similar number and diversity of BGCs, a higher number of NRPS was found (Fig. 5). One to six NRPS clusters were predicted from the selected *Streptomyces* species, representing an average of 4% of total BGCs, while 8 NRPS were identified in *S*. *fildesensis* So13.3 genome corresponding to a 19% of the total BGCs. NRPS are composed of multienzymes responsible for assembling complex linear and cyclic peptides, which can incorporate more than 500 different monomers including proteinogenic l-amino acids, non-proteinogenic l-amino acids, d-amino acids, modified l- and d-amino acids, α-hydroxy acids, and fatty acids^80^. Clusters 25, 27 and 28 from *S*. *fildesensis* So13.3 showed no similarities with known BGCs, however some might be part of other gene clusters as they are composed of one or two genes. Meanwhile NRPS clusters 21, 22, 23, 24 and 26 (Supplementary Table S1), had some similar genes with known secondary metabolites including the antibiotics actinomycin (25%), enduracidin (12%) and cinnamycin (19%); and the siderophore albachelin (50%). The clusters from So13.3 strain contain some of the biosynthetic genes from the known BGCs (see Supplementary Fig. S4), suggesting a possible functional and structural homology with the known antimicrobial molecules. The core structure from cluster 22 (similar to albachelin siderophore) and 24 (similar to cinnamycin antibiotic) were predicted by AntiSMASH, assuming molecules with molecular weights of 325.45 and 316.32, respectively. The latter was identified as a strong signal in the mass spectrum from the crude extract (Fig. 2a). A 325.45 m/z peak was not obtained from mass spectrum data, however, the gene cluster 24 includes multiple N-acetyltransferase genes and assuming a transfer of an acetyl group to the primary amine of the molecule we can predict an approximate 367.5 m/z signal, which is present as part of a metabolites cluster obtained from GNPS networking analysis (Fig. 2b). The result could suggest that metabolites found in network C2 from the crude extract (C2 in Fig. 2b) are encoded from the genetic cluster 24, producing multiple related intermediates from tailoring and biosynthesis process. As result the eight nodes on the molecular network represents structurally similar molecules with analogous fragmentation losses since they shared a common structural core. Mutagenesis and NMR analysis for chemical structure elucidation could confirm the aforementioned assumptions.

In addition, though melanins are commonly found in nature, the melanin BGC was only found in five of the 23 *Streptomyces* genomes, including our strain So13.3 (Fig. 5). Melanins are macromolecules ‒often brown colored pigments‒ produced by oxidative polymerization of phenolic or indolic compounds^81^. They play important roles in microorganisms against thermal, chemical, and biochemical stresses; and have important biological activities, including antimicrobial, antitumor, antivenin, anti-inflammatory and antioxidant^81^. Melanin cluster from *S*. *fildesensis* So13.3 showed similarity with 28% of the genes from the known melanin BGC, and no matches up to 1% were found with any other gene clusters on databases, including the melanins from the four *Streptomyces* related genomes considered in this study (*S*. *avermitilis*, *S*. *yerevanensis*, *S*. *auratus and S*. *roseoverticillatus*). Novelty and activity of melanin from So13.3 needs further analysis to be determined.

Similarly, about half of the taxonomically closest *Streptomyces* selected genomes (11) were found to have clusters encoding for lassopeptides (Fig. 5), including our strains So13.3. One cluster from So13.3 was predicted to encode an unknown lassopeptide with genes for carboxyvinyl-carboxyphosphonate, aldo/keto reductase, asparagine synthase and a putative macrolatam domain (GSSSSGNAD). Lassopeptides are a recently discovered group of natural compounds with diverse biological functions^82^. They are ribosomally synthesized and post-translationally modified peptides that contain a unique macrolactam ring motif. This structural feature confers high stability against proteolytic and chemical degradation, and exceptional thermal stability^82,83^. Therefore, lassopeptides are of great interest for antimicrobial activity.

Further chemical identification is necessary to confirm the possible new lassopeptide molecule and its activity from *Streptomyces* sp. So13.3. Although, the cluster show some similarity with clavulanic acid, it is unlikely that biosynthesis and final product has some homology with this metabolite, as the matches were recovered from two regulatory genes. Also, it is important to notice that this cluster seems to be highly regulated including four genes for transcriptional regulation (an AraC family member, an ArsR family member, a sensor histidine kinase and a response regulator), and four TTA codons close to the start codon of regulatory genes and biosynthetic genes. Expression of lassopeptide from So13.3 might require specific culture conditions and stimulation.

As expected, most of the gene clusters contain genes for transcriptional regulation, and interestingly 22 out of 42 gene clusters contain TTA codons. Among the six codons that encode leucine, UUA is very rare in *Streptomyces* as only 2–3% of the protein-coding genes contain TTA codons, which depend of the master regulator BldA, the only tRNA that read the UUA codon efficiently in *Streptomyces* genomes and responds to the secondary metabolisms^84,85^. Therefore, the presence of UUA codon suggests a highly‒regulated expression as part of the secondary metabolism. In addition, most abundant transcriptional regulator genes were found to belong to the LuxR family (12), SarP family (8), and TetR family (7), but others such as histidine kinase sensors, autoinducers, and transcriptional regulators from families ArsR, MerC, AraC, and AnsC were also found.

## Discussion

Among eight Antarctic *Streptomyces* isolates, the strain identified as *Streptomyces fildesensis* So13.3 showed the higher antimicrobial potential, showing a growth reduction against Gram‒negative pathogens and a remarkable MIC value against Gram‒positive pathogens. Studies have described MIC values >100 μg/mL in crude extracts from *Streptomyces* strains^86^, while our crude extract from *S*. *fildesensis* So13.3 showed a MIC value of 15.6 μg/mL for Gram-positive pathogens (Table 2), which is comparable with reported purified compounds ‒MIC values ranging from 2 to 75 μg/mL^55,87–89^ ‒ and with MIC value obtained from nalidixic acid positive control ranging from 2 to 8 μg/mL for susceptible strains (*E*. *coli* ATCC 22925 and *Klebsiella pneumoniae* ATCC 13883, respectively). This result implies that crude extract from *S*. *fildesensis* So13.3 is composed of one or more molecules with high antimicrobial activity against Gram‒positive bacteria and the MIC value from a purified extract could be predicted to be remarkably low. In addition, TLC bio-autography and mass spectrometry analysis allow us to conclude that antimicrobial molecules are present in the low polarity fractions of the *Streptomyces fildesensis* So13.3 crude extract, with molecular weight between 148 and 624 m/z. Since no matches were found with known molecules in GNPS library and only few BGCs were identified, the discovery of novel molecules from *S*. *fildesensis* So13.3 is expected. However, further characterization is required to confirm the novelty of these antibiotic molecules and to determine if a single compound contains all the antimicrobial activity or if several molecules with similar migration (by TLC) generate bacterial inhibition.

Genomic analysis of *S*. *fildesensis* So13.3 and whole genome comparison with close related species suggest that the diversity of BCGs on *Streptomyces* species might be dictated by recent HGT evolutionary events dependently of the environment conditions of each strain, since no evident correlations were found regarding the genetic distances and the BGCs compositions on the genomes. Some remarkable differences were found on the *S*. *fildesensis* So13.3 genome characterization. The first one is that considering all predicted and putative BGCs, *S*. *fildesensis* So13.3 devoted a 22% (2.05 Mb) of its genome content to secondary metabolites productions. It has been established that the average species BGCs content is 3.7% ± 3.1% of the genome, and some *actinobacteria* was defined outlier species with >7.5% of their genomes dedicated to natural product biosynthesis considering the mean fraction of a bacterial genome devoted to transcription (7.2%) and translation (8.5%)^90^. Only one species from the latter work (*Streptomyces bingchenggensis*) showed a comparable 22% of its genome devoted to secondary metabolites^90^. The second one is the higher content of NRPS BGCs, and the presence of lassopeptides, melanins which were less frequently found on the close related species. Therefore, lassopeptides, melanins and NRPS from *S*. *fildesensis* So13.3 are of great interest for antimicrobial activity and drug discovery.

Particularly, PKS, NRPS, PKS/NRPS hybrid, bacteriocin, lantipeptide and lassopeptides gene clusters are of particular attention in the search for novel antibiotic families in light of the worrisome trends in the occurrences of antimicrobial resistant human pathogens^91^. The abundance of low similarity or unknown BGCs from the above types in the genome of *S*. *fildesensis* So13.3, as well as its broad range of antimicrobial activity against Gram‒positive bacteria, including MRSA, demands further research efforts to identify the encoded metabolites that could lead to the discovery of multiple potential bioactive molecules. Although some BGCs showed high similarity with known biosynthetic genes, BGCs from *S*. *fildesensis* So13.3 appear to have interesting extra genes that might lead to modification on the biosynthetic pathway, changing the molecule and its activity. This was the case for BGCs identified with 100% similarity. Extra biosynthetic and transport genes were found on ectoine and 2‒methylisoborneol genetic cluster, where, and particularly, alkylresorcinol cluster from So13.3 contains several additional biosynthetic, transporter and regulatory genes including the drug resistance transporter EmrB/QacA, a multi‒drug efflux pump responsible for the export of toxic molecules, which could be a possible mechanism to resist self‒antimicrobial production, as described for other novel antibiotics molecules like the benzoxazole caboxamycin^92^. Moreover, this cluster includes multiple acetyltransferases, oxidoreductases, methyltransferases, an esterase, a hydrolase and a chalcone and stilbene synthase. As known for PKS III, those enzymes catalyze elongation of diverse acyl-CoA starter units with one or more malonyl-CoA extender units to form poly-β-ketoacyl-CoA intermediates that undergo a range of cyclization reactions to form diverse aromatic products^93^; and specifically chalcone and stilbene synthases have been reported in some bacteria and plants as a type III PKSs responsible for the production of germicidin in *S*. *coelicolor*, flaviolin in *S*. *venezuelae*, and 1,3,6,8-tetrahydroxynaphthalene (THN) in several *Streptomyces* species^94^.

Our results suggest that it is possible ‒as it has been speculated‒ that adaptation to the Antarctic environment confers different genetic features to *S*. *fildesensis* So13.3, which was evidenced by the high content of BGCs with low or any similarities with known metabolites. As evidence, melanin cluster, which is commonly found in nature, were found to be virtually unique for *S*. *fildesensis* So13.3, with less that 1% with the genetic clusters on databases. More interesting, high transcriptional regulation of BGCs from *S*. *fildesensis* So13.3 suggest the fine tune of the bacterial response for environmental signaling by the production of these secondary metabolites. With this in mind, it is expected that even the annotated BGCs from *S*. *fildesensis* So13.3 might be producing different molecules and possible new drugs.

Besides the aforementioned findings, all 16 putative BGCs matching with known gene cluster showed similarities with biosynthetic genes from known antibiotics. These molecules may possess some structural or functional analogy to the predicted putative BGC. Our work highlights the antimicrobial nature of *S*. *fildesensis* So13.3 and the potential of its genome as reservoir for drug discovery. Additional *in vitro* and genomic analysis should be undertaken to confirm the presence, expression and novelty of secondary metabolites found in *S*. *fildesensis* So13.3 genome sequences, as well as to elucidate the nature of their antimicrobial activity. Functional genomics, RNA-seq and heterologous expression will be a future study to confirm responsible DNA for the antimicrobial activity.

## Supplementary information

Antarctic Streptomyces fildesensis So13.3 strain as a promising source for antimicrobial discovery

## Data Availability

All data generated or analyzed during this study are included in this published article and its Supplementary Information files.
