# Supplementary material for: Antarctic Streptomyces fildesensis So13.3 strain as a promising source for antimicrobials discovery
Source: Sci Rep. 2019 May 16;9:7488. doi: 10.1038/s41598-019-43960-7 (PMC6522549; doi:10.1038/s41598-019-43960-7)
Supplement: Supplementary file 1 — Antarctic Streptomyces fildesensis So13.3 strain as a promising source for antimicrobial discovery [file 41598_2019_43960_MOESM1_ESM.docx]

**Antarctic *Streptomyces fildesensis* So13.3 strain as a promising source for antimicrobials discovery**

Kattia Núñez-Montero^1, 2, 3^, Claudio Lamilla^1, 2^, Michel Abanto^2^, Fumito Maruyama^2, 4^, Milko A. Jorquera^2, 5^, Andrés Santos^1,2,6^, Jaime Martinez-Urtaza^6^, Leticia Barrientos^1, 2^*

*^1^ Laboratorio de Biología Molecular Aplicada, Centro de Excelencia en Medicina Traslacional, Universidad de La Frontera, Temuco, Chile*

*^2^ Núcleo Científico y Tecnológico en Biorecursos (BIOREN), Universidad de La Frontera, Temuco, Chile*

*^3^ Centro de Investigación en Biotecnología, Escuela de Biología, Instituto Tecnológico de Costa Rica, Cartago, Costa Rica.*

*^4^ Department of Microbiology, Graduate School of Medicine, Kyoto University, Yoshida‒Konoe‒cho, Sakyo‒ku, Kyoto, Japan*

*^5^ Laboratorio de Ecología Microbiana Aplicada, Departamento de Ciencias Químicas y Recursos Naturales, Universidad de La Frontera, Temuco, Chile*

*^6^ Centre for Environment, Fisheries and Aquaculture Science (CEFAS), Barrack Road, Weymouth, Dorset DT4 8UB, UK*

*****Correspondence:

Dr. Leticia Barrientos

E‒mail address: leticia.barrientos@ufrontera.cl

Tel: (+56) 45‒2592802.

**Supplementary Table S1.** Gene clusters for secondary metabolites biosynthesis of Antarctic *Streptomyces fildesensis* So13.3.

| Cluster | Type | Length (bp) | Most similar known cluster (% gene similarity) | MIBiG BGC-ID^a^ |
| --- | --- | --- | --- | --- |
| Cluster 1 | Bacteriocin | 10848 | - | - |
| Cluster 2 | Bacteriocin | 11907 | Daptomycin (3%) | BGC0000336_c1 |
| Cluster 3 | Bacteriocin-T1PKS | 60365 | Maduropeptin (3%) | BGC0001008_c1 |
| Cluster 4 | Butyrolactone- Fatty Acid-T1PKS | 65777 | RK-682 (54%) | BGC0000140_c1 |
| Cluster 5 | Fatty Acid | 23046 | - | - |
| Cluster 6 | Fatty Acid | 21289 | Feglymycin (21%) | BGC0001233_c1 |
| Cluster 7 | Fatty Acid | 13696 | - | - |
| Cluster 8 | Fatty Acid-NRPS | 36158 | Myxochelin (16%) | BGC0001345_c1 |
| Cluster 9 | Fatty Acid-T1PKS | 54199 | Marineosin (86%) | BGC0000091_c1 |
| Cluster 10 | Fatty Acid-T1PKS | 28385 | RK-682 (36%) | BGC0000140_c1 |
| Cluster 11 | Saccharide | 41533 | - | - |
| Cluster 12 | Saccharide | 18639 | Glycopeptidolipid (5%) | BGC0000362_c1 |
| Cluster 13 | Saccharide | 26710 | - | - |
| Cluster 14 | Ectoine | 10452 | Ectoine (100%) | BGC0000853_c1 |
| Cluster 15 | Lantipeptide | 22687 | Polyoxin (5%) | BGC0000877_c1 |
| Cluster 16 | Lantipeptide | 22756 | SapB (100%) | BGC0000551_c1 |
| Cluster 17 | Lantipeptide-Terpene | 56485 | Lipopeptide 8D1-1 (9%) | BGC0001370_c1 |
| Cluster 18 | Lassopeptide | 22628 | Clavulanic acid (8%) | BGC0000845_c1 |
| Cluster 19 | Melanin | 10422 | Melanin (28%) | BGC0000908_c1 |
| Cluster 20 | Melanin-Saccharide | 34223 | Istamycin (2%) | BGC0000700_c1 |
| Cluster 21 | NRPS | 23873 | Actinomycin (25%) | BGC0000296_c1 |
| Cluster 22 | NRPS | 62943 | Albachelin (50%) | BGC0001211_c1 |
| Cluster 23 | NRPS | 43386 | Enduracidin (12%) | BGC0000341_c1 |
| Cluster 24 | NRPS | 30706 | Cinnamycin (19%) | BGC0000503_c1 |
| Cluster 25 | NRPS | 9610 | - | - |
| Cluster 26 | NRPS | 3661 | Actinomycin (7%) | BGC0000296_c1 |
| Cluster 27 | NRPS | 2180 | - | - |
| Cluster 28 | NRPS | 1856 | - | - |
| Cluster 29 | Other | 31396 | Actinomycin (75%) | BGC0000296_c1 |
| Cluster 30 | Other | 32060 | A-503083 (3%) | BGC0000288_c1 |
| Cluster 31 | Other | 24673 | Borrelidin (4%) | BGC0000031_c1 |
| Cluster 32 | Siderophore | 14875 | Scabichelin (20%) | BGC0000423_c1 |
| Cluster 33 | Siderophore | 14711 | - | - |
| Cluster 34 | T1PKS | 24320 | Lankamycin (20%) | BGC0000085_c1 |
| Cluster 35 | T1PKS | 2795 | - | - |
| Cluster 36 | T1PKS-NRPS | 36135 | Cremimycin (22%) | BGC0000042_c1 |
| Cluster 37 | T2PKS | 38905 | Lysolipin (52%) | BGC0000242_c1 |
| Cluster 38 | T2PKS | 60861 | Spore pigment (83%) | BGC0000271_c1 |
| Cluster 39 | T3PKS | 57126 | Alkylresorcinol (100%) | BGC0000282_c1 |
| Cluster 40 | Terpene | 21100 | - | - |
| Cluster 41 | Terpene | 11047 | 2-methylisoborneol (100%) | BGC0000658_c1 |
| Cluster 42 | Terpene | 18065 | Hopene (76%) | BGC0000663_c1 |
| Cluster 43 | Putative | 24282 | - | - |
| Cluster 44 | Putative | 18643 | - | - |
| Cluster 45 | Putative | 24572 | - | - |
| Cluster 46 | Putative | 20274 | - | - |
| Cluster 47 | Putative | 5175 | - | - |
| Cluster 48 | Putative | 16498 | Laspartomycin (4%) | BGC0000379_c1 |
| Cluster 49 | Putative | 19896 | A47934 (17%) | BGC0000290_c1 |
| Cluster 50 | Putative | 8260 | - | - |
| Cluster 51 | Putative | 25571 | - | - |
| Cluster 52 | Putative | 25303 | - | - |
| Cluster 53 | Putative | 8350 | - | - |
| Cluster 54 | Putative | 44230 | - | - |
| Cluster 55 | Putative | 28349 | Streptomycin (19%) | BGC0000717_c1 |
| Cluster 56 | Putative | 13331 | Kijanimicin (4%) | BGC0000082_c1 |
| Cluster 57 | Putative | 20860 | - | - |
| Cluster 58 | Putative | 10602 | - | - |
| Cluster 59 | Putative | 13300 | - | - |
| Cluster 60 | Putative | 6220 | - | - |
| Cluster 61 | Putative | 13622 | Chondrochloren (11%) | BGC0000970_c1 |
| Cluster 62 | Putative | 8316 | Novobiocin (20%) | BGC0000834_c1 |
| Cluster 63 | Putative | 21569 | - | - |
| Cluster 64 | Putative | 5475 | - | - |
| Cluster 65 | Putative | 44812 | - | - |
| Cluster 66 | Putative | 8924 | - | - |
| Cluster 67 | Putative | 20564 | - | - |
| Cluster 68 | Putative | 21566 | - | - |
| Cluster 69 | Putative | 10763 | - | - |
| Cluster 70 | Putative | 31632 | Lomofungin (13%) | BGC0001302_c1 |
| Cluster 71 | Putative | 6754 | - | - |
| Cluster 72 | Putative | 7364 | - | - |
| Cluster 73 | Putative | 16403 | - | - |
| Cluster 74 | Putative | 14699 | - | - |
| Cluster 75 | Putative | 9518 | - | - |
| Cluster 76 | Putative | 9844 | - | - |
| Cluster 77 | Putative | 7321 | - | - |
| Cluster 78 | Putative | 9767 | - | - |
| Cluster 79 | Putative | 36123 | Thiotetronate Tu 3010 (5%) | BGC0001352_c1 |
| Cluster 80 | Putative | 6503 | Kanamycin (1%) | BGC0000703_c1 |
| Cluster 81 | Putative | 12090 | Kanamycin (15%) | BGC0000704_c1 |
| Cluster 82 | Putative | 4739 | - | - |
| Cluster 83 | Putative | 12829 | Neocarzinostatin (6%) | BGC0000112_c1 |
| Cluster 84 | Putative | 26922 | Lomofungin (13%) | BGC0001302_c1 |
| Cluster 85 | Putative | 12765 | JBIR-34, JBIR-35 (8%) | BGC0000376_c1 |
| Cluster 86 | Putative | 11096 | Dactylocycline (5%) | BGC0000216_c1 |
| Cluster 87 | Putative | 42026 | - | - |
| Cluster 88 | Putative | 4800 | - | - |
| Cluster 89 | Putative | 11834 | - | - |
| Cluster 90 | Putative | 10730 | - | - |
| Cluster 91 | Putative | 8500 | - | - |
| Cluster 92 | Putative | 7541 | - | - |
| Cluster 93 | Putative | 28369 | - | - |
| Cluster 94 | Putative | 13442 | - | - |
| Cluster 95 | Putative | 15191 | Platencin (6%) | BGC0001156_c1 |
| Cluster 96 | Putative | 6942 | Lactonamycin (3%) | BGC0000238_c1 |
| Cluster 97 | Putative | 10005 | - | - |
| Cluster 98 | Putative | 4988 | - | - |

^a^ The Minimum Information about a Biosynthetic Gene Cluster (Genomic Standards Consortium).

**Supplementary Figure S1.** Growth determinations for *Streptomyces* sp. strains isolated from Antarctic soil. Growth kinetics of the eight Antarctic *Streptomyces* strains tested for antimicrobial activity. μ= specific growth rate for each strain. Error bars = standard deviation.

**Supplementary Figure S2.** Growth rate reduction of Gram‒negative pathogens after 20 h culture exposed to different concentrations of *Streptomyces* sp. So13.3 crude extract. Growth rate was calculated as the absorbance (600nm) at 20h treatment divided by the absorbance at 0h treatment. Significant differences (ANOVA) from crude extract concentrations were presented in all the three Gram‒negative pathogens tested (**p* ≤ 0.01) at concentrations greater than 31μg/mL

**Supplementary Figure S3.** Similarity graphics of metabolites from *Streptomyces fildesensis* So13.3 compared to reference clusters for ectoine, SapB, alkylresorcinol and 2‒methylisoborneol. *Streptomyces fildesensis* So13.3 cluster compare to reference cluster from Biosynthetic Gene Cluster at Genomic Standards Consortium. Colored tags (blue, red, yellow and green) show the specific genes match for the known metabolite biosynthesis. Additional related, biosynthetic, regulatory and transport genes are shown.

**Supplementary Figure S4.** Predicted gene clusters and core structures for NRPS clusters 22 and 24 from *Streptomyces fildesensis* So13.3 compared to reference clusters for the siderophore albachelin and the antibiotic cinnamycin. Colored tags show the specific genes matches with known metabolite biosynthesis. Additional related, biosynthetic, regulatory and transport genes are shown in white. Chemical structures are a prediction of core scaffold based on assumed NRPS collinearity, tailoring reactions not considered.

**Supplementary Figure S5.** Quality report of trimmed sequences obtained from the whole genome sequencing of *Streptomyces fildesensis* So13.3 by Illumina HiSeq X sequencing platform. Sequence lengths and quality score distribution over all sequences are shown for the pair-end library.
